# Quality of life in octogenarians with non small cell lung cancer: the strategic role of video assisted thoracic surgery

**DOI:** 10.1186/1471-2318-11-S1-A65

**Published:** 2011-08-24

**Authors:** N D Vicidomini, G Guggino, G Monaco

**Affiliations:** 1 Unità di Chirurgia Toracia, Università degli Studi di Napoli Federico II, Napoli, Italy; 2 Unità Operativa di Chirurgia Toracica, AORN Antonio Cardarelli, Napoli, Italy

## Background

The aim of the present study was to assess the surgical approach in octogenarians comparing the benefits of video-assisted thoracic surgery (VATS) with the open thoracotomy and analysing early and long term quality of life (QOL) changes.

## Materials and methods

We reviewed 42 consecutive octogenarians (mean age 82.3±1.4 years) with a preoperative FEV1 of 1.5 L or less who had undergone pulmonary resection for stage I non-small cell lung cancer (NSCLC). Patients were divided into two groups according to surgical approach (VATS group n=22; thoracotomy group n=20) and their postoperative complications and prognoses were evaluated. Quality of life was assessed using Medical Outcome Study Short-Form 36-item Health Survey (SF-36) just before surgery and at 6 and 12 months postoperatively.

## Results

Morbidity rate and postoperative hospital stay were significantly different between VATS and thoracotomy group (p=0.01; p=0.0003, respectively). A worse decrease in thoracotomy vs VATS group was demonstrated in five domains at 6 months, and in all domains at 12 months of SF-36 questionnaire. The thoracotomy group presented a significant reduction in the dyspnoea index, FEV1 and DLCO at both 6 and 12 months.

## Conclusions

In octogenarians with NSCLC and compromised pulmonary function the long-term survival justifies the operative treatment, and minimally invasive surgical approach enables surgeons to extend pulmonary resection in these high-risk patients if selected appropriately.
